# A Rare Presentation of Five Primary Cancers in a Patient With Werner Syndrome: A Case Report and Literature Review

**DOI:** 10.7759/cureus.77534

**Published:** 2025-01-16

**Authors:** Ruchi Yadav, Shakthi Raman, Dosbai Saparov, Vivek Yadav, Avezbakiyev Boris

**Affiliations:** 1 Hematology/Oncology, Brookdale University Hospital Medical Center, Brooklyn, USA; 2 Internal Medicine, Medical University of Lublin, Lublin, POL; 3 Internal Medicine, Brookdale University Hospital Medical Center, Brooklyn, USA; 4 Pulmonary and Critical Care Medicine, South Georgia Medical Center, Valdosta, USA

**Keywords:** dna damage and repair, premature aging, primary cancers, werner syndrome, wrn gene

## Abstract

Werner syndrome (WS) is a rare autosomal recessive disorder characterized by premature aging and a higher cancer risk. WS patients are characterized by a defective gene product, WRN, that plays an instrumental role in the genomic stability of DNA structures. It typically manifests in late adolescence or early adulthood, leading to premature aging, age-related disorders like diabetes, and myocardial infarction, and an increased propensity for developing sarcomas, melanoma, and solid tissue cancer. We present a rare case of an 80-year-old female with WS developing five different primary cancers over a decade namely basal cell carcinoma (BCC) of the face, left-sided urothelial carcinoma, right-sided triple-negative breast cancer (TNBC), right-sided invasive colonic adenocarcinoma, and pancreatic intraductal papillary mucinous neoplasm (IPMN). Our case report is unique in presentation it defies the expected life expectancy of the 50s seen in WS, with the patient currently exhibiting a stable clinical course. This case highlights the need for further research into the mechanisms behind extended lifespan and atypical tumor spectra in such patients, as well as the development of tailored therapeutic strategies, particularly with regard to chemotherapy.

## Introduction

Werner syndrome (WS) was first described in 1904 by German physician Otto Werner [1] as a rare autosomal recessive disorder caused by mutations in the WRN gene notable for its hallmark feature of premature aging, in association with other comorbidities, including malignancies [2]. Notably, its prevalence in the United States is reported as one in 200,000 live births, with increased frequency in certain areas of the world, including Japan, Sardinia, Pakistan, and India [2,3]. The spectrum of the clinical manifestations of WS depicts bilateral cataracts, premature graying and loss of hair, scleroderma-like skin changes, and a distinctive “bird-like” facial appearance [2]. Additionally, the risk of developing cancer in WS is significantly elevated compared to the general population including osteosarcomas, melanomas, and thyroid carcinomas [3]. Our case report is atypical in the presentation of WS as our patient is in the eighth decade of her life surviving multiple morbid diseases and myriads of primary cancers. This patient, therefore, exceeds the typical life expectancy of those with WS by more than two decades, adding a unique dimension to her clinical picture.

## Case presentation

We present an extremely rare case of an 80-year-old female with WS and developing five distinct primary types of cancer including basal cell carcinoma (BCC) in 2010 with recurrence in 2019, left-sided urothelial carcinoma in 2018, right-sided triple-negative breast cancer (TNBC) in 2020, right-sided invasive colonic adenocarcinoma in 2023, and pancreatic intraductal papillary mucinous neoplasm (IPMN) in 2024. Her medical history includes hypertension, type 2 diabetes mellitus, atrial fibrillation, gastroesophageal reflux disease, hyperlipidemia, osteoporosis, asthma, macrocytic anemia, and chronic kidney disease (CKD) stage 3B. Her family history is significant for early-stage cervical cancer in her daughter and for diabetes and hypertension in her mother and sister. She reports that she has never smoked or used smokeless tobacco. She also reports that she does not drink alcohol or use illicit drugs.

Basal cell carcinoma

At 66 years of age, she was diagnosed with high-risk BCC of the right cheek, in 2010, and underwent Mohs surgery. After surgery, the margins were negative and were under active surveillance. However, in 2019 she developed a recurrence/lesion at the same location, pathologically diagnosed as BCC, and subsequently managed with standard excision with wide surgical margins and healing by reconstruction of the skin flap on the right side of the face. Unfortunately, as the patient received treatment at an outside facility around 14 years ago, only the clinical note was available for reference, without any pathology slides.

High-grade left papillary urothelial carcinoma

In May 2018, at 74 years of age, the patient presented with gross hematuria, and a contrast-enhanced CT scan of the abdomen/pelvis demonstrated a left renal pelvis mass measuring 2.3 x 1.5 cm. She had a series of procedures including a renal pelvis biopsy, fulguration, ureteral stent implantation, cystoscopy, ureteroscopy, and pyeloscopy. The pathology resulted in high-grade papillary urothelial carcinoma in the ureter and renal pelvis with no stromal invasion. One month later in August 2018, she underwent a robotic-assisted left distal ureterectomy with partial cystectomy, left nephrectomy, and para-aortic lymph node dissection. Pathological diagnosis showed invasive high-grade papillary urothelial carcinoma, with areas of inverted growth and concomitant urothelial carcinoma in situ with involvement of von Brunn nests. The tumor measured 1.4 cm in greatest dimension (gross size) and involves the renal pelvis and ureteropelvic junction. Multifocal invasion of lamina propria (pT1) presents invasion with all four lymph nodes negative for carcinoma. No angiolymphatic invasion was identified. Surgical margins were negative for carcinoma. The kidney showed benign renal parenchyma with multiple cortical cysts. Based on her final pathological report of pT1N0M0, she was recommended surveillance with scans, cystoscopy, and urine cytology at regular intervals. During surveillance, she remained stable, with no significant new abnormalities identified on repeat cystoscopy, cytology, and imaging scans. The patient received treatment for the above-mentioned cancer at an outside facility around six years ago; therefore, only the clinical note was available for reference, without any imaging pictures or pathology slides.

Triple-negative infiltrating ductal carcinoma of the right breast

**Figure 1:**
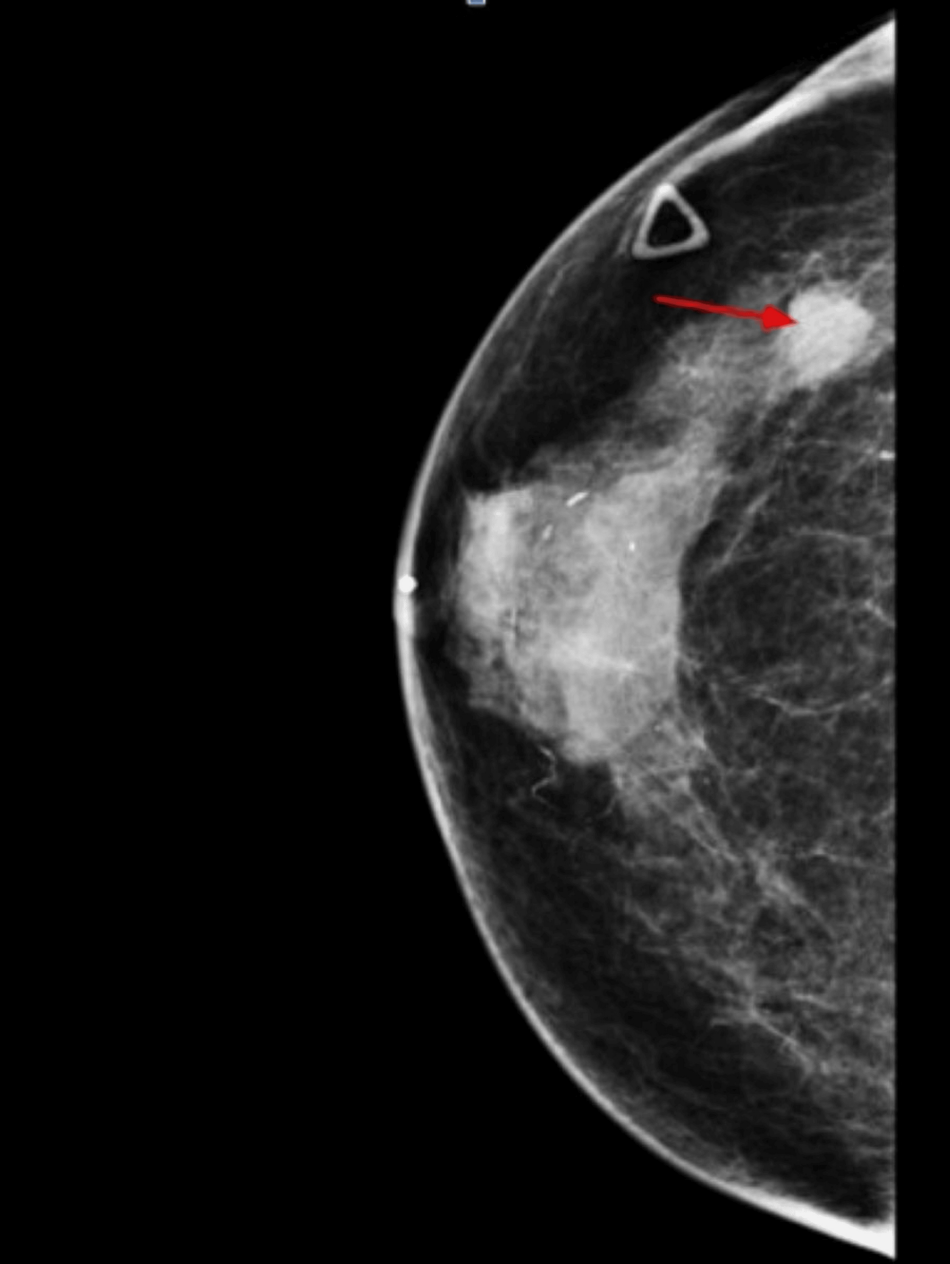
Mammogram of the right breast showing a transversely palpable marker overlying the right upper outer quadrant. There is nodule and 1.4 cm mass, which persists on spot compression at approximately 10-11 o'clock position (red arrow).

**Figure 2:**
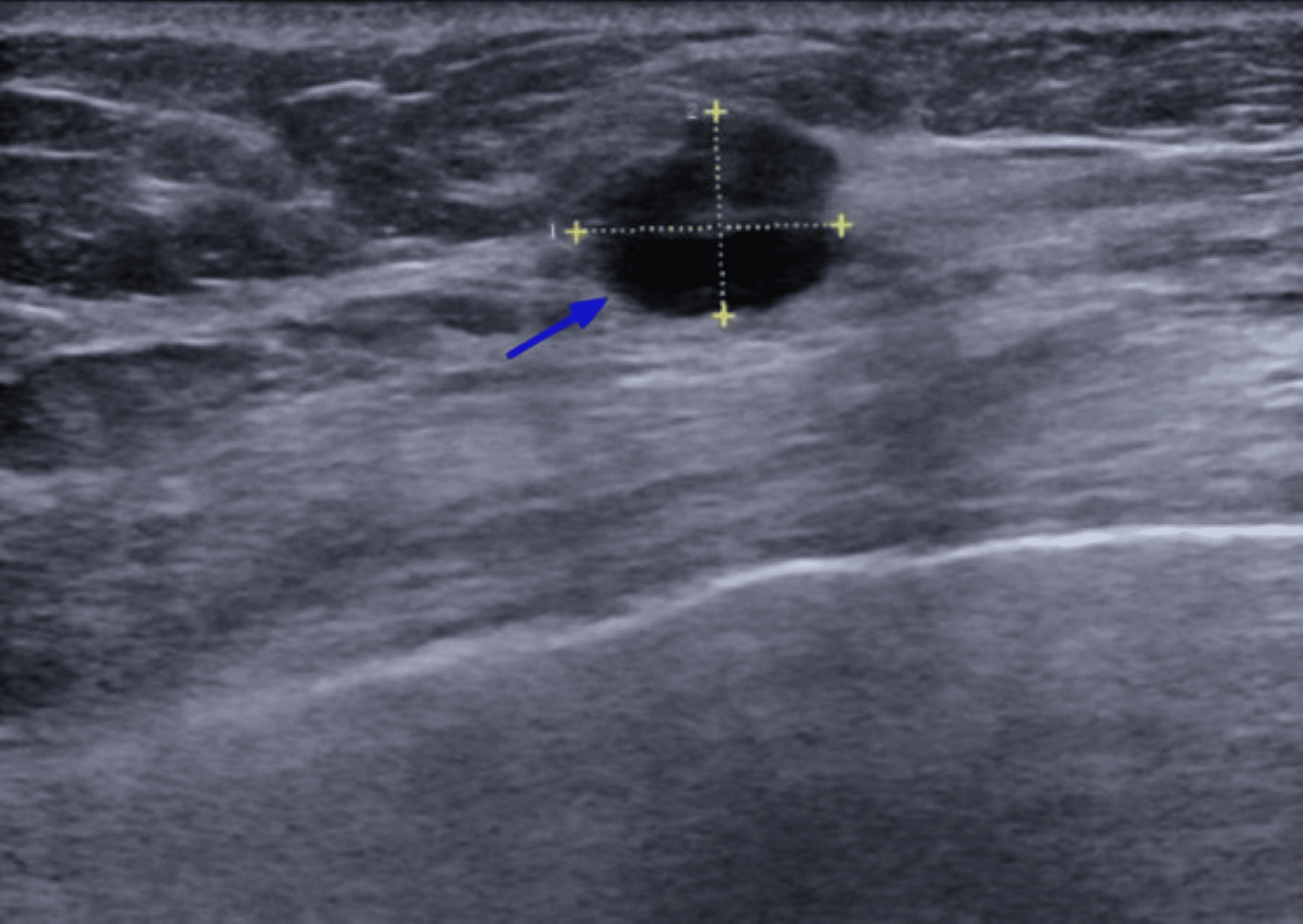
Ultrasound of the right breast showed a 1.2 x 0.9 x 1.4 cm suspicious age-related mass (blue arrow) at 10-11 o'clock position, 5 cm from the nipple. Both axillae are imaged and are normal.

The patient noted a palpable mass in her right breast in September 2020 with subsequent mammogram (Figure 1) and sonogram (Figure 2) of the bilateral breasts confirming the presence of a right breast mass at the 10-11 o'clock position.

Biopsy of the breast mass resulted in pathology revealing infiltrating ductal carcinoma of the right breast with estrogen receptor (ER), progesterone receptor (PR), and HER2 receptor negative (triple negative). Based on AJC TNM clinical staging, she was diagnosed with stage 1B infiltrating ductal carcinoma of the breast. She underwent a right mastectomy and sentinel lymph node biopsy of the axillary lymph nodes in November 2021, with pathological staging showing lymph nodes negative for tumor. The invasive ductal adenocarcinoma was 1.5 x 1 x 1 cm, moderately to poorly differentiated, with a Nottingham score of 7 (2+3+2). ER/PR/HER2 FISH was negative/not amplified, and the Ki-67 proliferative index was reported as 25-30%.

She received adjuvant chemotherapy with adriamycin 60 mg/m² and cyclophosphamide 600 mg/m² (AC) every three weeks for four cycles, followed by weekly paclitaxel 80 mg/m² for 12 weeks. She completed her treatment in April 2021. Follow-up diagnostic mammograms and ultrasound imaging as part of active surveillance showed benign findings with no evidence of malignancy.

In the context of three different primary cancers, sequence analysis, and deletion/duplication testing of the 84 genes listed in the Genes Analyzed section were performed in May 2022. A heterozygous pathogenic variant, Exon 9, c.1105C>T (p.Arg369*), was identified in WRN. This sequence change creates a premature translational stop signal (p.Arg369) in the WRN gene. It is expected to result in an absent or disrupted protein product. Loss-of-function variants in WRN are known to be pathogenic. This premature translational stop signal has been observed in individual(s) with WS. The method of genetic testing includes the following: Genomic DNA obtained from the submitted sample is enriched for targeted regions using a hybridization-based protocol and sequenced using Illumina technology. Unless otherwise indicated, all targeted regions are sequenced with ≥50x depth or are supplemented with additional analysis. Reads are aligned to a reference sequence (GRCh37), and sequence changes are identified and interpreted in the context of a single clinically relevant transcript. Enrichment and analysis focus on the coding sequence of the indicated transcripts, 20 bp of flanking intronic sequence, and other specific genomic regions demonstrated to be causative of disease at the time of assay design. Promoters, untranslated regions, and other non-coding regions are not otherwise interrogated. For some genes only targeted loci are analyzed. Exonic deletions and duplications are detected using an in-house algorithm that determines the copy number at each target by comparing the read depth for each target in the proband's sequence with the mean read depth and read-depth distribution obtained from a set of clinical samples. Markers across the X and Y chromosomes are analyzed for quality control purposes and may detect deviations from the expected sex chromosome complement. Such deviations may be included in the report in accordance with internal guidelines. Confirmation of the presence and location of reportable variants is performed based on stringent criteria established by Invitae as needed, using one of several validated orthogonal approaches. 

Proximal ascending colon invasive adenocarcinoma

**Figure 3:**
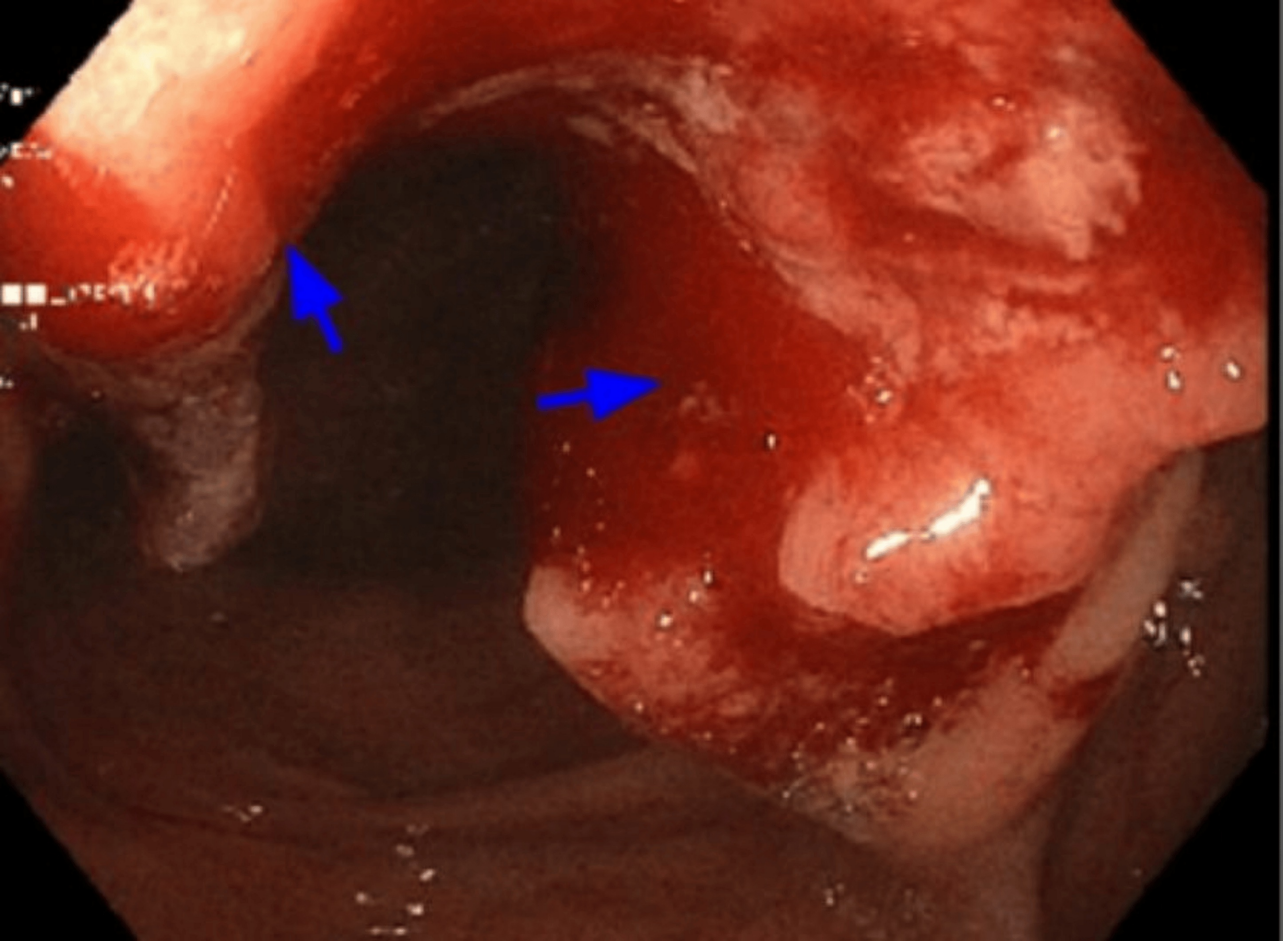
A semi-circumferential, non-obstructive, ulcerated, friable mass measuring approximately 4 x 6.5 cm was found in the proximal ascending colon, with overlying friable and ulcerated mucosa, during colonoscopy (blue arrows).

In May 2023, the patient had a positron emission tomography (PET) scan as part of surveillance for previous history of left-sided urothelial carcinoma. An incidental finding of intensely FDG-avid mass-like thickening in the proximal ascending colon and a probable smaller nodule in the cecum, suspicious for malignancy, was noted. In July 2023, she underwent a diagnostic colonoscopy that showed a semi-circumferential (65% extent), ulcerated, friable mass measuring approximately 4 x 6.5 cm at the proximal ascending colon, with overlying friable and ulcerated mucosa (Figure 3).

**Figure 4:**
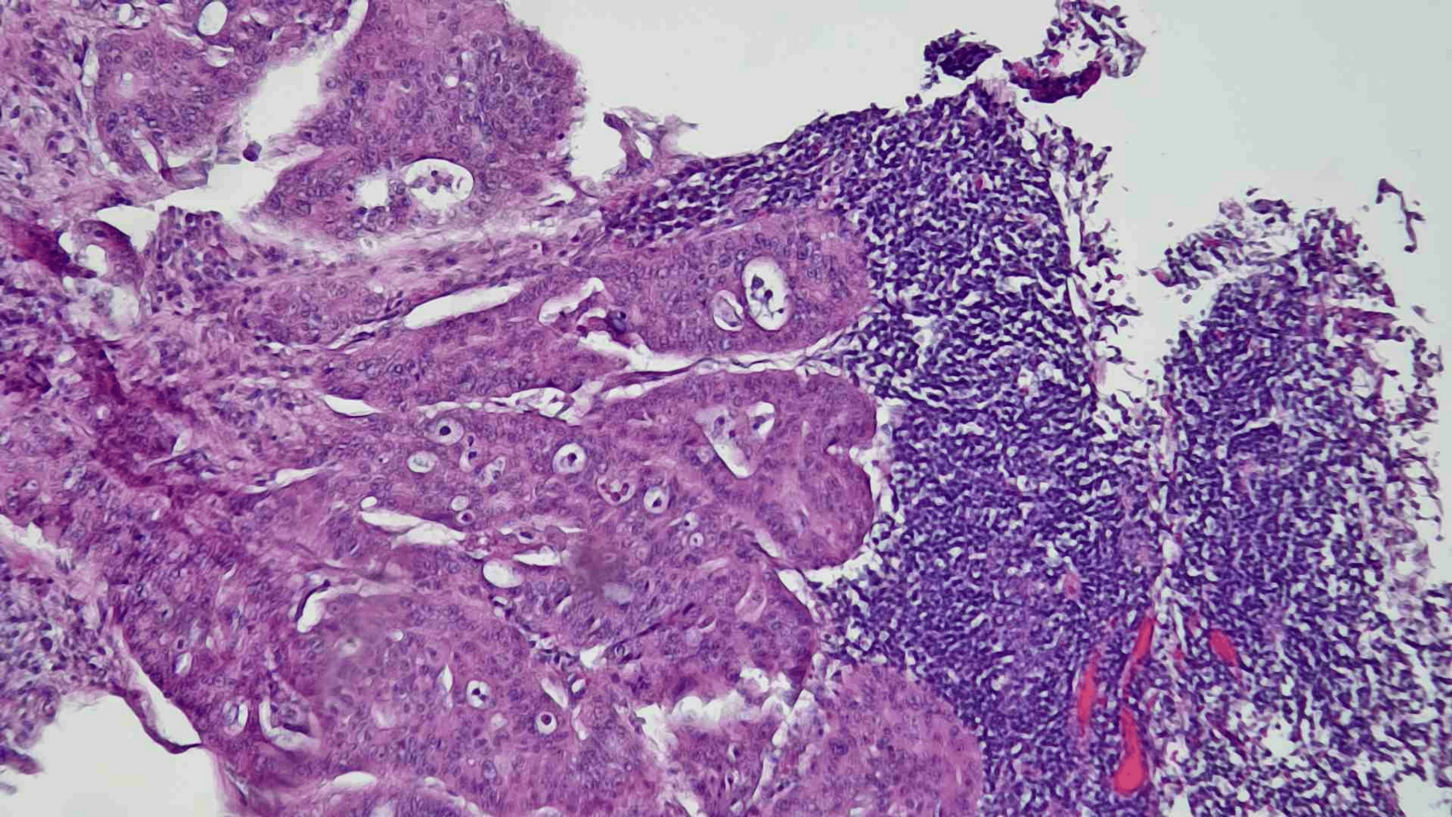
Hematoxylin & eosin staining in 100x magnification showing metastasis in lymph node with invasive colonic adenocarcinoma.

**Figure 5:**
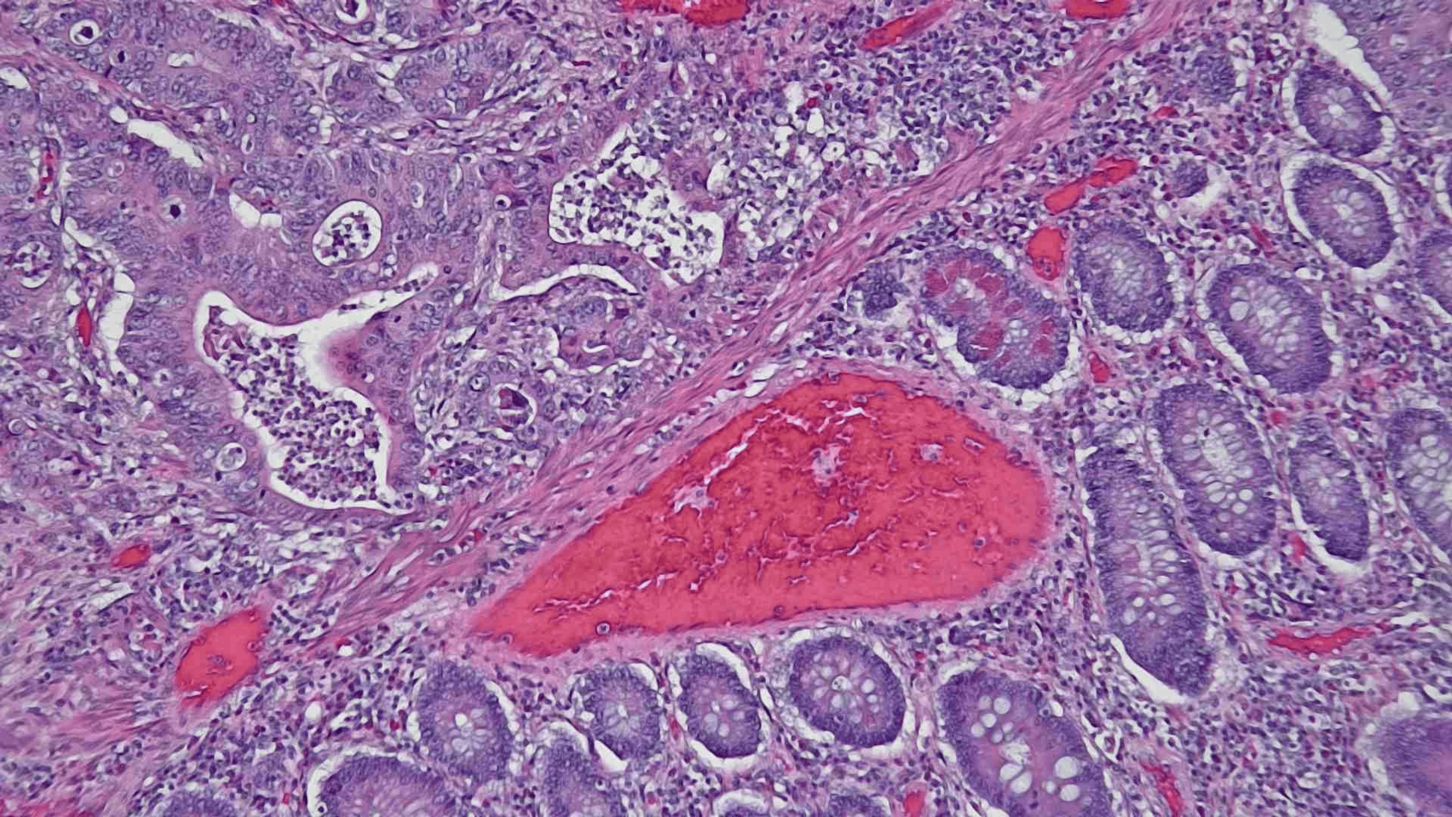
Hematoxylin & eosin staining in 100x magnification showing invasive colonic adenocarcinoma with adjacent benign appearing mucosa.

Multiple cold forceps biopsies were obtained. The colonic mucosa appeared normal at the cecum, ileocecal valve, appendiceal orifice, in the rest of the ascending colon, hepatic flexure, transverse colon, splenic flexure, descending colon, sigmoid colon, and rectum. The result of the surgical pathology showed invasive colonic adenocarcinoma, moderately differentiated. For further staging, CT chest/abdomen/pelvis with contrast showed a concentric mass in the ascending colon consistent with the findings of colonoscopy with no evidence of any distant metastasis. She was referred to a colorectal surgeon for further opinion and underwent a laparoscopic right hemicolectomy in October 2023. The final surgical pathology report showed invasive adenocarcinoma of the colon (Figure 4 and Figure 5) with moderate- to high-grade differentiation, measuring 4 x 3.5 x 2 cm. The carcinoma was invasive into the peri-colonic adipose tissue (pT3), with negative resection margins (R0). Focal evidence of thin-walled vessel involvement by the carcinoma was noted, and two out of 21 lymph nodes were positive for adenocarcinoma (pN1). The final staging was pT3N1M0, stage III.

Immunohistochemical (IHC) staining showed no loss of nuclear expression of MMR (mismatched repair) proteins and a low probability of microsatellite instability. Her Eastern Cooperative Oncology Group (ECOG) Performance Status was 1. She began capecitabine 1500 mg twice a day for six months as part of adjuvant therapy. Serial monitoring and surveillance, including history and physical examination, carcinoembryonic antigen (CEA), circulating tumor DNA (ctDNA), and CT imaging, showed a good response with no evidence of tumor.

Pancreatic intraductal papillary mucinous neoplasm

**Figure 6:**
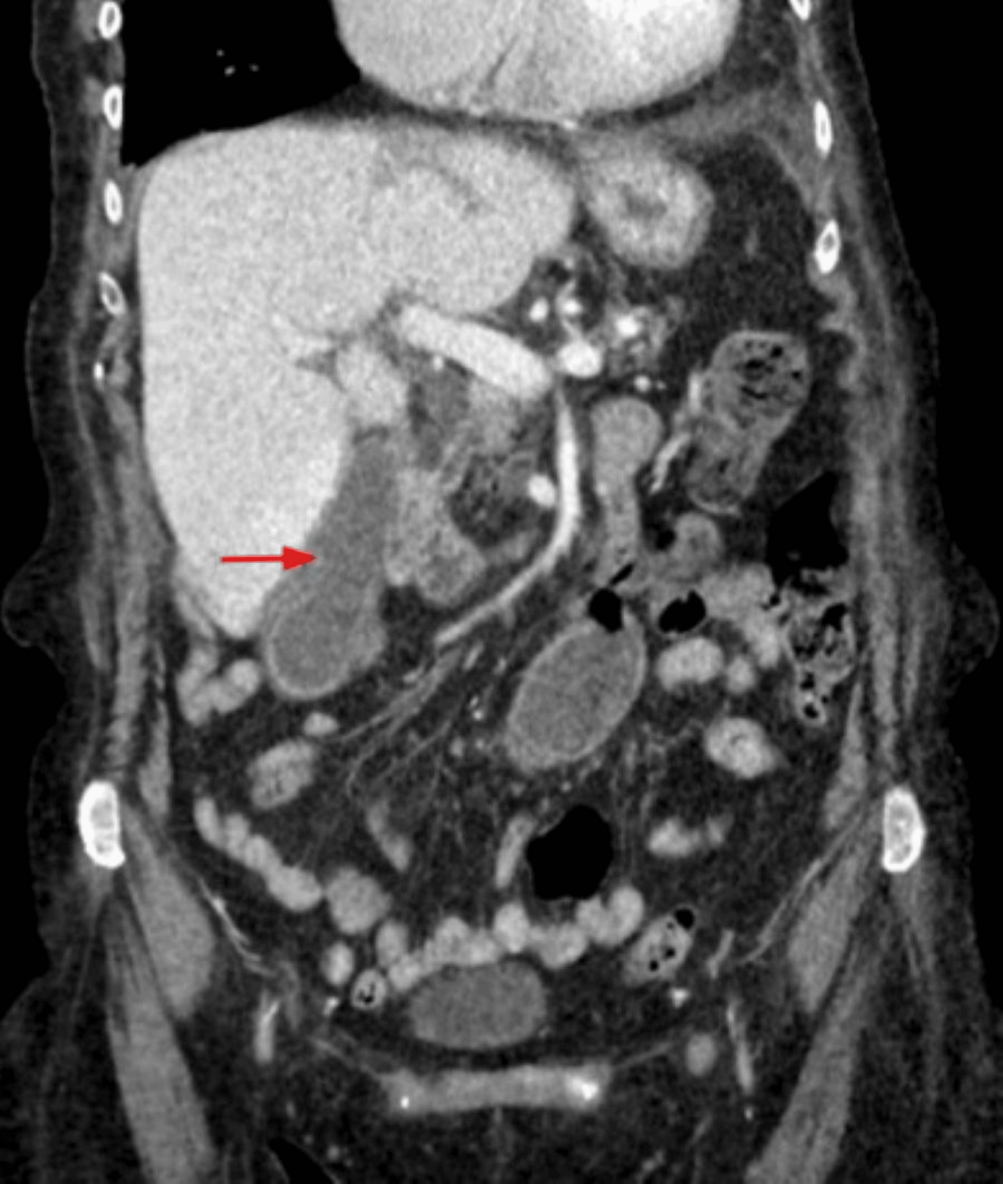
CT abdomen and pelvis with contrast showing a moderately distended gallbladder with mild wall enhancement and pericholecystic inflammatory changes (red arrow). Acute cholecystitis cannot be excluded.

The patient was admitted in May 2024 with signs and symptoms of acute cholecystitis. A CT scan of the abdomen and pelvis with contrast (Figure 6) showed pericholecystic inflammatory changes.

**Figure 7:**
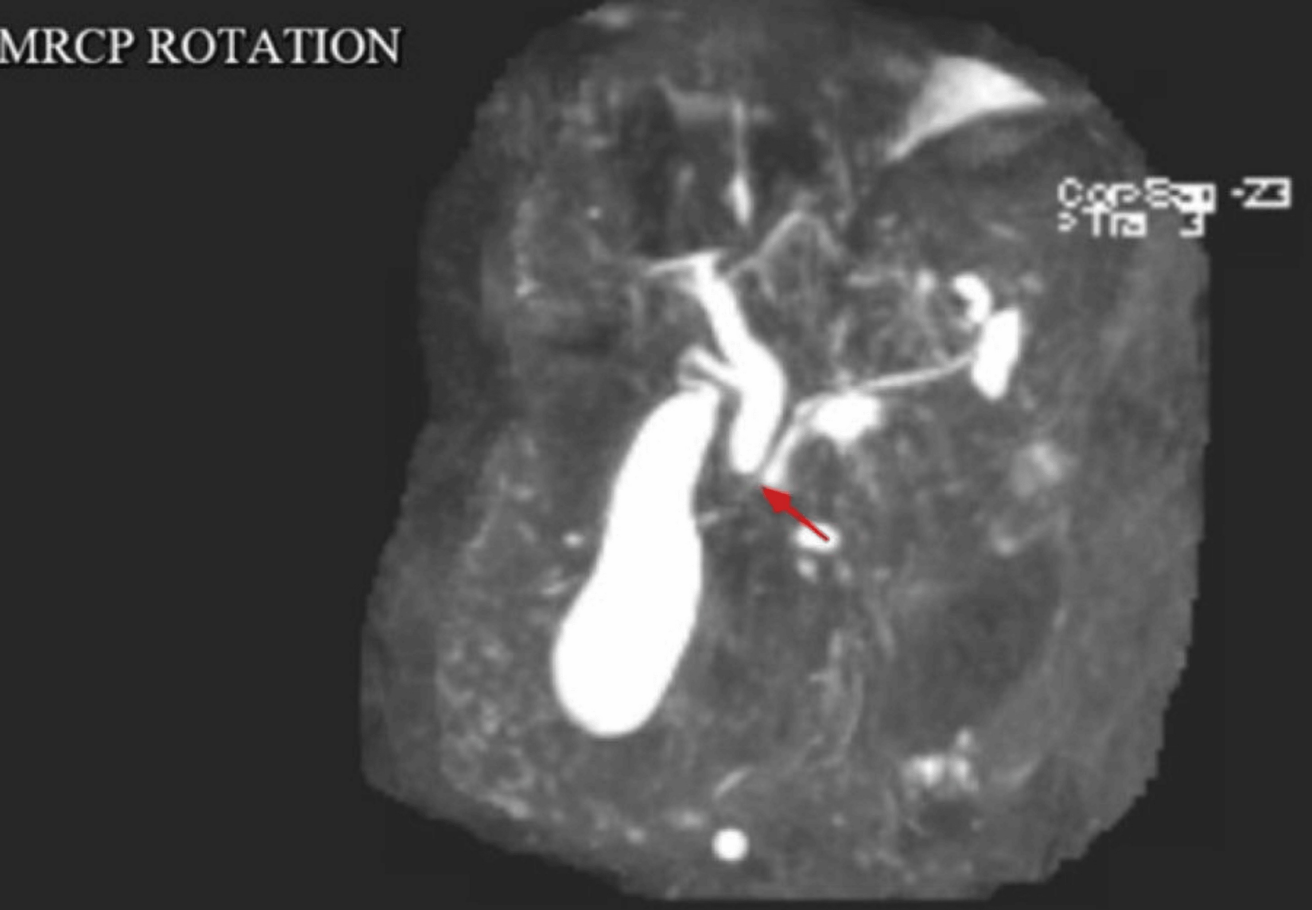
An MRCP image showing mild dilatation of the CBD measuring 8 mm with an abrupt cutoff of the common duct just proximal to the ampulla, which likely represents some internal sludge or small ductal stone or inflammatory stricture (red arrow). CBD, common bile duct; MRCP, magnetic resonance cholangiopancreatography

Magnetic resonance cholangiopancreatography (MRCP) revealed an 8 mm dilated common bile duct (CBD) with an abrupt cutoff, suggestive of a possible small duct stone, biliary sludge, and a potential inflammatory stricture (Figure 7).

**Figure 8:**
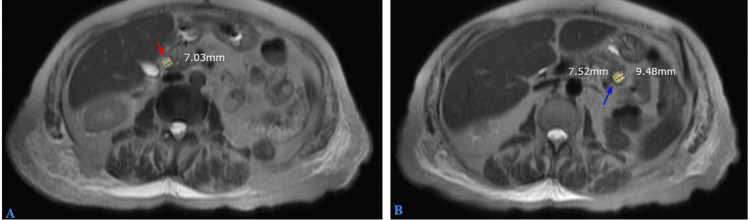
MRI with a pancreatic protocol showed the pancreas to be mildly atrophic with mild prominence of the pancreatic duct. A cyst was noted in the pancreatic head (A, red arrow) and an additional cyst in the pancreatic tail (B, blue arrow), likely representing a small IPMN. MRI, magnetic resonance imaging; IPMN, pancreatic Intraductal papillary mucinous neoplasm

Magnetic resonance imaging (MRI) with a pancreatic protocol showed the pancreas to be mildly atrophic with mild prominence of the pancreatic duct. A cyst was identified in the pancreatic head (Figure 8A, red arrow) and an additional cyst in the pancreatic tail (Figure 8B, blue arrow), likely representing a small IPMN.

**Figure 9:**
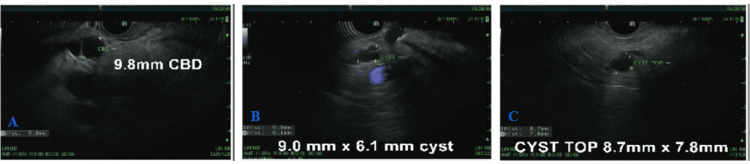
EUS showing CBD dilation to 9.8 mm (A). No stones, sludge, strictures, masses, or abrupt cutoffs were noted in the CBD; therefore, ERCP was not performed. Additionally, two anechoic cysts were found: one in the pancreatic head and the other in the pancreatic body, measuring 9.0 x 6.1 mm and 8.7 x 7.8 mm, respectively (B and C). EUS, endoscopic ultrasound; CBD, common bile duct; ERCP, endoscopic retrograde cholangiopancreatography

The patient was evaluated by a gastroenterologist (GI) specialist and underwent an endoscopic ultrasound (EUS) showing CBD dilation to 9.8 mm(Figure 9A). There were no stones, sludge, strictures, masses, or abrupt cutoffs in the CBD; therefore, no endoscopic retrograde cholangiopancreatography (ERCP) was performed. Additionally, two anechoic cysts were found: one in the head and the other in the body of the pancreas, measuring 9.0 x 6.1 mm and 8.7 x 7.8 mm, respectively (Figures 9B, 9C). No fine needle aspiration/biopsy was performed on the pancreatic cysts due to their small size and deep location within the pancreas. The final diagnosis was consistent with a likely side branch IPMN. As per records, the patient refused any further workup and agreed to regular surveillance and monitoring for the same.

## Discussion

The case of this 80-year-old female with WS, who has developed five distinct primary malignancies over 14 years, presents a rare and complex clinical presentation that has not been reported before. WS, first described by German physician Otto Werner in 1904, is a rare autosomal recessive disorder caused by mutations in the WRN gene notable for its hallmark feature of premature aging, in association with other comorbidities, including malignancies [1,2]. The incidence of WS is estimated to be between the range of <1 per 100,000 births to one per 10,000 in general with prevalence in the United States reported as one in 200,000 live births, with increased frequency in certain areas of the world, including Japan, Sardinia, Pakistan, and India [3].

**Figure 10:**
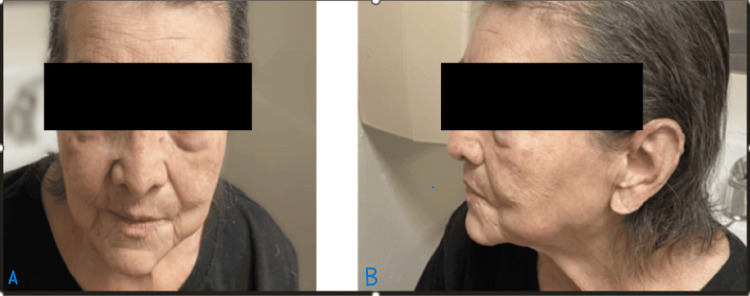
Typical facial features of WS, including bird-like facies, thin lips, and early aging, as evident in the real photo of the patient with identifying information hidden. (A) Front profile. (B) Lateral profile.

Clinically, WS patients often develop normally during childhood and adolescence, with signs of the disorder typically emerging in the second or third decade of life, differentiating WS from other progeroid syndromes that manifest earlier [4]. One of the first noticeable features is the lack of a growth spurt during adolescence, resulting in short stature, a consistent characteristic among WS patients [1,5]. The clinical manifestations of WS are multisystemic. Patients often present with bilateral cataracts, premature graying and loss of hair, scleroderma-like skin changes, and a distinctive “bird-like” facial appearance, including a beaked nose and thin lips [1,6]. Ocular changes, along with the characteristic facial features evident in our patient (Figure 10A, front profile; Figure 10B, lateral profile), often prompt the initial clinical suspicion of WS.

Additionally, individuals with WS are predisposed to a range of age-related conditions. Musculoskeletal manifestations of WS include osteoporosis, and metabolically patients frequently develop type 2 diabetes mellitus, often before the age of 40 [6,7]. They also show an increased predisposition to dyslipidemia and insulin resistance, leading to the accelerated onset of atherosclerosis and cardiovascular disease [1]. As a result, cardiovascular complications are one of the leading causes of death in WS patients, often occurring in the fourth or fifth decade of life [6,7]. Our case report is atypical in presentation as our patient is in the eighth decade of her life after surviving multiple morbid diseases and cancers. This patient, therefore, exceeds the typical life expectancy of those with WS by more than two decades, adding a unique dimension to her clinical picture.

WS results from biallelic mutations in the WRN located on chromosome 8p12, which typically acts to encode a member of the ReQ helicase family, a group of enzymes that are essential for the maintenance of genomic stability through their involvement in DNA replication, recombination, and repair [7]. As the WRN protein plays a pivotal role in several DNA metabolic pathways including telomeric maintenance, a nonfunctional WRN may result in telomere shortening and chromosomal end-to-end fusions, both of which may contribute to cellular senescence and premature aging as observed in WS patients [7,8]. Moreover, WRN-deficient cells exhibit defective DNA double-strand break (DSB) repair, an inability to resolve stalled replication forks, and an increased frequency of chromosomal aberrations, including deletions and translocations, which significantly heighten cancer risk in these individuals. For this reason, malignancies are considered a critical feature of WS [7].

The risk of developing cancer in WS is significantly elevated compared to the general population, with tumors often presenting at earlier ages [6,7]. The spectrum of cancers in WS is broad, with frequent reports of osteosarcomas, melanomas, and thyroid carcinomas [2]. Malignancies are the second leading cause of death in WS patients, after cardiovascular complications [5], though it has been noted that morbidity due to malignancies is increasing [9].

The myriads of different cancer presentations in our patient are unique compared to the spectrum of malignancies seen in other reported cases of WS. A pathogenic variant, c.1105C>T (p.Arg369), that was identified in WRN led to the genetic instability associated with her mutated WRN protein likely contributing to the development of multiple malignancies including urothelial carcinoma, BCC, TNBC, colonic adenocarcinoma, and pancreatic IPMN. Urothelial carcinoma, diagnosed in this patient in 2018, is notable given the frequency of epithelial cancers in WS2. Although not as common as sarcomas and thyroid cancers, urothelial carcinoma has been previously reported in WS patients, often attributed to impaired DNA repair mechanisms in rapidly dividing cells [2].

The development of BCC of the skin the following year is not surprising, as WS is associated with skin cancers, typically basal or squamous cell carcinomas, though higher frequency was noted for melanomas [2,10]. Interestingly, however, it has not been evidenced that WRN-deficient cells exhibit a particular sensitivity to factors such as ultraviolet radiation, despite the aforementioned possibility of deficient double-stranded break repair that could be induced [11]. The patient’s age at the time of BCC diagnosis is atypical, as such malignancies usually present earlier in WS, suggesting the potential involvement of environmental factors or differences in genetic expression within WS subtypes.

The diagnosis of TNBC in this patient in 2020 is additionally significant, as WS patients have an increased risk for breast cancer, particularly hormone receptor-negative subtypes like TNBC [2]. Given that TNBC is aggressive and lacks targeted hormonal treatments, it is a considerable therapeutic challenge, and its development highlights the aggressive oncogenic potential in WS patients.

Colonic adenocarcinoma of the proximal ascending colon, diagnosed in 2023 in this patient, is consistent with the increased risk of gastrointestinal cancers in WS [2]. Proximal colon cancers, specifically, have a more aggressive clinical course and poorer prognosis; however, in our case, good clinical response to surgery and adjuvant therapy was noted. The genetic instability caused by the WRN mutation likely contributed to the patient's susceptibility to this malignancy, as WS patients often have defects in both homologous recombination and mismatch repair systems, exacerbating their risk for gastrointestinal cancers [2]. In 2024, the patient was diagnosed with pancreatic IPMN, a rare but critical malignancy associated with pancreatic cancer precursors. Pancreatic malignancies are not commonly seen in WS, but given the patient’s established history of multiple primary cancers and the underlying genetic basis, this diagnosis highlights the extensive oncogenic burden in WS patients [2].

The diagnostic process for WS itself is primarily based on clinical recognition of its characteristic dermatologic, ophthalmologic, and metabolic features [12]. Genetic testing to identify pathogenic mutations in the WRN gene is the gold standard for diagnosis [5]. Early detection is critical, as it enables the implementation of surveillance strategies aimed at reducing morbidity and mortality from cancer and cardiovascular diseases in WS patients as well as at improving quality of life [4]. This case also highlights the potential benefits of genetic counseling for families affected by WS. Given the autosomal recessive inheritance of the WRN mutation, genetic screening of relatives may help identify carriers and inform them about their cancer risks and potential need for surveillance.

In terms of management, the case underscores the necessity of vigilant cancer screening in WS patients. Given the patient’s history of multiple primary malignancies, early detection and intervention have likely played a crucial role in her survival. Regular screening for malignancies, particularly in organs where WS patients are at higher risk, such as the gastrointestinal tract, skin, and breast tissue, remains a cornerstone of patient care. Moreover, the use of personalized therapeutic approaches, potentially targeting DNA repair pathways, could offer promising avenues for managing WS-associated malignancies in the future [2].

Chemotherapy for patients with WS can be particularly challenging. As the genomic instability of WS stems from defects in the DNA repair process, these patients may have an increased susceptibility to the toxicities of chemotherapy, which often works by damaging the DNA of malignant cells to induce cell death. The inherent DNA repair defects may complicate treatment, as the cells may either be hypersensitive to DNA damage or they may develop resistance mechanisms more easily, depending on the chemotherapeutic agent employed [11].

WRN-deficient cells display significant hypersensitivity to DNA-damaging agents, particularly those that induce interstrand crosslinks, such as cisplatin, mitomycin C, and melphalan [13]. In addition, these cells are also vulnerable to 4-nitroquinoline-1-oxide, certain alkylating agents, and topoisomerase I inhibitors like camptothecin [11]. Our patient was exposed to a course of cyclophosphamide as part of TNBC treatment and interestingly responded well without any documented toxicities.

Furthermore, WRN-deficient cells show increased sensitivity to hydroxyurea (HU) and aphidicolin, which do not induce direct DNA damage but rather impair replication by depleting nucleotide pools or inhibiting DNA polymerases [14]. This suggests that the WRN protein plays a critical role in managing cellular responses to replication stress or stalling [11]. Supporting this idea, apoptosis in WRN-deficient cells following these treatments primarily occurs during the S or G2 phase of the cell cycle, when replication and DNA damage repair mechanisms are actively engaged [14].

Some studies have indicated that WRN deficiency may lead to chemoresistance under certain conditions [15]. The mechanisms of chemoresistance in WS are likely multifactorial, involving changes in drug metabolism, upregulation of detoxifying enzymes, and modulation of apoptotic pathways. For example, increased expression of glutathione-related enzymes may contribute to resistance to cisplatin by neutralizing the drug before it can induce sufficient DNA damage to kill cancer cells​ [16,17].

In a study involving WRN-deficient lymphoblastoid cell lines (LCLs), it has been demonstrated that these cells did not undergo a differential apoptotic response compared to wild-type cells in response to intercalating drugs, such as daunomycin and adriamycin [18]. This data suggests that the WRN helicase/exonuclease may not be directly involved in the removal or repair of DNA intercalating agents and that these medications may be affected to a lesser extent by WS than other chemotherapeutic agents.

The patient’s ability to reach the age of 80, despite her WS diagnosis, suggests potential mitigating factors, such as a favorable genetic variation, aggressive medical management, or lifestyle factors that may have contributed to her prolonged survival. Her longevity in the face of such a significant cancer burden further underscores the variability in disease presentation and progression in WS, making it a rare and valuable case for understanding the spectrum of WS pathology.

## Conclusions

What makes this case particularly unique is that the patient has lived well beyond the average life expectancy for individuals with WS, which typically ranges between the late 40s to mid-50s due to the early onset of cardiovascular diseases or malignancies. This case adds to the growing body of literature demonstrating the significant cancer predisposition in WS. The development of five distinct malignancies diagnosed over 14 years in an individual well beyond the typical age of death for WS highlights both the intrinsic genetic factors as well as the extrinsic biological environment playing a significant role in survival. This case reinforces the need for ongoing research into the underlying pathological and genetic etiologies of WS facilitating an early diagnosis and treatment including screening protocols for this set of patient population.
